# Results from early programmatic implementation of Xpert MTB/RIF testing in nine countries

**DOI:** 10.1186/1471-2334-14-2

**Published:** 2014-01-02

**Authors:** Jacob Creswell, Andrew J Codlin, Emmanuel Andre, Mark A Micek, Ahmed Bedru, E Jane Carter, Rajendra-Prasad Yadav, Andrei Mosneaga, Bishwa Rai, Sayera Banu, Miranda Brouwer, Lucie Blok, Suvanand Sahu, Lucica Ditiu

**Affiliations:** 1 Stop TB Partnership, Secretariat, Geneva, Switzerland; 2 Interactive Research and Development, Karachi, Pakistan; 3 Pôle de Microbiologie, Institut de Recherche Expérimentale et Clinique (IREC), Université Catholique de Louvain (UCL), Brussels, Belgium; 4 Health Alliance International, University of Washington, Seattle, WA, USA; 5 University of Wisconsin, Madison, WI, USA; 6 Project HOPE, Mulanje, Malawi; 7 Warren Alpert School of Medicine at Brown University, Providence, RI, USA; 8 Stop TB Unit, WHO, Phnom Penh, Cambodia; 9 Center for Health Policies and Studies, Chisinau, Republic of Moldova; 10 International Organization for Migration, Kathmandu, Nepal; 11 Tuberculosis & Leprosy Research Group; Centre for Communicable Diseases; International Centre for Diarrhoeal Disease Research, Bangladesh, Dhaka, Bangladesh; 12 PHTB Consult, Tilburg, Netherlands; 13 KIT Development Policy & Practice, Royal Tropical Institute, Amsterdam, Netherlands

**Keywords:** New diagnostics, Xpert MTB/RIF, Tuberculosis, Case detection, Innovation

## Abstract

**Background:**

The Xpert MTB/RIF assay has garnered significant interest as a sensitive and rapid diagnostic tool to improve detection of sensitive and drug resistant tuberculosis. However, most existing literature has described the performance of MTB/RIF testing only in study conditions; little information is available on its use in routine case finding. TB REACH is a multi-country initiative focusing on innovative ways to improve case notification.

**Methods:**

We selected a convenience sample of nine TB REACH projects for inclusion to cover a range of implementers, regions and approaches. Standard quarterly reports and machine data from the first 12 months of MTB/RIF implementation in each project were utilized to analyze patient yields, rifampicin resistance, and failed tests. Data was collected from September 2011 to March 2013. A questionnaire was implemented and semi-structured interviews with project staff were conducted to gather information on user experiences and challenges.

**Results:**

All projects used MTB/RIF testing for people with suspected TB, as opposed to testing for drug resistance among already diagnosed patients. The projects placed 65 machines (196 modules) in a variety of facilities and employed numerous case-finding strategies and testing algorithms. The projects consumed 47,973 MTB/RIF tests. Of valid tests, 7,195 (16.8%) were positive for MTB. A total of 982 rifampicin resistant results were found (13.6% of positive tests). Of all tests conducted, 10.6% failed. The need for continuous power supply was noted by all projects and most used locally procured solutions. There was considerable heterogeneity in how results were reported and recorded, reflecting the lack of standardized guidance in some countries.

**Conclusions:**

The findings of this study begin to fill the gaps among guidelines, research findings, and real-world implementation of MTB/RIF testing. Testing with Xpert MTB/RIF detected a large number of people with TB that routine services failed to detect. The study demonstrates the versatility and impact of the technology, but also outlines various surmountable barriers to implementation. The study is not representative of all early implementer experiences with MTB/RIF testing but rather provides an overview of the shared issues as well as the many different approaches to programmatic MTB/RIF implementation.

## Background

Each year an estimated 8.6 million people worldwide develop tuberculosis disease (TB), yet fewer than 6 million cases are reported and treated [1]. In addition, of the more than 300,000 estimated multidrug-resistant TB cases among all notified cases, less than a third were reported in 2012 [1]. One of the impediments to improving case detection and early initiation of treatment for both drug-sensitive and-resistant TB is the limited availability of sensitive and rapid TB diagnostics.

In December 2010, the World Health Organization (WHO) endorsed the use of the Xpert MTB/RIF® assay (MTB/RIF) for the detection of TB and rifampicin resistance [2]. The assay uses a closed cartridge that simply requires insertion into a GeneXpert machine, with similar infection control laboratory interventions as those for smear microscopy [3]. These characteristics allow placement of the system at lower level health facilities (e.g. district or sub-district facilities), which should theoretically improve access to testing. A multi-country demonstration study documented high levels of sensitivity and specificity to detect *Mycobacterium tuberculosis* bacteria and rifampicin resistance [4], while a recent Cochrane systematic review supported the initial demonstration study’s findings [5]. WHO recommends using the MTB/RIF assay as a primary diagnostic test for persons suspected of having a DR-TB or in settings with high HIV prevalence and, where resources permit, as a second test for those with an abnormal chest x-ray (CXR) and/or negative smear microscopy results [2].

Globally, there has been significant interest in using the MTB/RIF test across a variety of settings, with over 2.3 million Xpert® MTB/RIF cartridges procured in the public sector through March 2013 [6]. Various studies have documented use of the MTB/RIF test in people with signs and symptoms of TB, on clinical specimens [7-11], and for the detection of drug resistance [12,13]. Several commentaries, cost-effectiveness studies and models have presented potential uses for and likely impacts of this assay [14-21]. However, there is a dearth of published literature produced by implementers of the technology in programmatic settings. As National TB Programs (NTPs) and their partners scale up the use of MTB/RIF tests for case detection, there is a need to share results from implementation in programmatic settings, especially since there is evidence that research studies and product inserts often provide a more optimistic view of test performance, as compared to real-world implementation [22].

TB REACH, a global initiative of the Stop TB Partnership [23], has supported many partners with GeneXpert technology through its competitive grant making process. We present results from nine TB REACH interventions, review the main challenges experienced and formulate recommendations for other early implementers.

## Methods

During the period from September 2011 to March 2013, a total of 30 TB REACH grants across 24 countries designated funds to procure GeneXpert machines and the Xpert MTB/RIF assay. Of these, a convenience sample of nine projects was selected for inclusion in this study with the aim of covering a range of implementers, regions and approaches. Standard quarterly reports from projects were utilized to analyze MTB/RIF testing data for patient yields, rifampicin resistance patterns and failed tests. The proprietary files were directly exported from the GeneXpert Dx software to verify data in quarterly reports. The data presented reflect the first twelve months of MTB/RIF assay implementation for each project. The start date of each project was different; however all data were collected between October 2011 and March 2013.

All projects completed an electronic questionnaire covering procurement and deployment experiences, diagnostic algorithms, training requirements, and recording and reporting issues. The questionnaire was followed up by semi-structured interviews with staff from each project either in person or over the phone. Interviews with the manufacturer were also conducted, addressing aspects of procurement. Questionnaire and interview data were analyzed for major themes across different aspects of MTB/RIF assay implementation.

Records utilized were from standard NTP data collected in the course of routine programmatic implementation with no personal identifiers. Therefore ethical review for this analysis was not sought. Individual projects in The Democratic Republic of Congo (DRC), Kenya, Pakistan, Bangladesh and Mozambique had waivers for their projects. The remaining projects in Cambodia, Malawi and Nepal were being implemented by or on behalf of NTPs as part of routine operations and did not seek ethical review.

## Results

### Placement and utilization

The projects were often the first implementers of the MTB/RIF test for TB case detection in their respective countries. Table 1 provides an overview of the selected nine TB REACH projects including location and case finding strategy. Placements included public and private hospitals and lower primary care facilities, private diagnostic laboratories, HIV centres, prisons, reference laboratories and mobile units. The projects were able to run the machines at district hospitals and at lower levels of care although in only a few situations were peripheral microscopy centres included, mostly because of throughput concerns. Additional files 1,2,3,4,5,6,7,8,9 show pictures of a number of the places where MTB/RIF was used. A number of interventions initially projected low MTB/RIF testing numbers due to the high cost of assay cartridges and the fact that the test was a new technology. In these instances, less expensive and lower-capacity 2-module machines were procured rather than standard 4-module units (see Table 2 for recommendations).

**Table 1:** Overview of early Xpert MTB/RIF test placement and TB case finding strategy in nine countries

**Country**	**WHO Country classifications**	**Placement**	**Number of GeneXpert machines**		**Case finding approach**
			**2-Module**	**4-Module**	
Bangladesh	HBC, HDR	Two urban private diagnostic laboratories	2		Systematic screening of all attendees
Cambodia	HBC	Both machines used at one-day mobile camps set up at primary care facilities		2	Active with promotion of testing days in community
DR Congo	HBC, HDR, HTH	One primary care center, one sub-district hospital, five district hospitals and one provincial laboratory.	7	1	Primarily passive but with employed community groups to refer people with suspected TB
Kenya	HBC, HTH	Two district hospitals and one health center.		3	Mixed, employed community cough workers to augment numbers of people tested
		All had ART delivery			
Malawi	HTH	One central hospital, four district hospitals, one health center in a district with no district hospital, one mission hospital and one community hospital.		8	Passive
Moldova	HDR	17 in public facilities at lowest level for TB diagnosis, two in regional AIDS centers, two in the national reference laboratory, two in the regional reference laboratory, and two in the penitentiary sector	16	9	Passive
Mozambique	HBC, HTH	Four district hospitals		4	Passive
Nepal	HBC	Four regional hospitals (two public and two private), one district hospital, two primary health centers, one private referral center, and one reference laboratory	3	6	Primarily passive with some awareness raising/and educational activities to improve numbers of people tested
Pakistan	HBC, HDR	Four urban private diagnostic laboratories	4		Systematic screening of all attendees

HBC = High Burden Country, HDR = High MDR-TB Burden Country, HTH = High TB/HIV Burden Country.

**Table 2:** Challenges and recommendations for programmatic implementation of Xpert MTB/RIF testing

**Implementation area**	**Challenges**	**Recommendations**
**Machine utilization**	Overestimation of testing numbers and underutilization of machine capacity.	Conduct a needs assessment that includes the current number of people with suspected TB tested at the facility, the need for a referral system and the impact of the proposed testing algorithm on testing numbers.
		Two-module machines may be a less expensive alternative in many settings.
**Testing algorithms**	Lack of national guidance on testing algorithms and use of questionable testing strategies.	To improve yield and reduce cartridge use, consider screening with CXR.
		Consider elimination of smear microscopy as a first test to reduce delay, loss to follow-up, and avoid repeat testing of most individuals due to large proportion of smear negative results.
**Time to diagnosis**	Sputum transport systems and testing algorithms can prolong delay between sputum submission, results and treatment initiation.	Xpert MTB/RIF testing sites should be located as close to the patient as possible to allow for rapid treatment initiation keeping in mind throughput and electrical power limitations.
		Referral networks can help utilization rates but can also be costly to maintain.
**Procurement**	Short shelf life of cartridges, use of machine beyond date of needed calibration, unanticipated extra costs of shipping and customs clearance, local procurement agent not always helpful.	Stagger cartridge shipments to avoid stock outs and expiry.
		Plan for module calibration by ordering test kits early.
		Plan for extra costs associated with shipment and customs clearance.
**Training**	Staff rotation and new practices around request forms, specimen transport and clinical decisions for rifampicin resistant results.	Testing can be easily done by well-trained lay workers to support laboratory staff.
		The manufacturer has conducted web trainings via videoconference and webinars and has released a web-based training video which some projects used as an adjunct tool for facilitation of trainings.
**Infrastructure and power supply**	UPS (15 minutes) Cepheid offers is inadequate in many settings. Most laboratories need some infrastructure improvements to allow proper testing.	A standard 800-2000VA inverter and a 12 V/120-200 AH battery provided power for over 6 hours to one four-module GeneXpert machine and a laptop (200 wattage required). As a general rule, a 10AH battery will be able to power a requirement of 100 watts per hour, but more AH are needed when the discharge is over a short period of time (less than 20 hours). These can be procured locally.
**Failed tests**	Differences between types of failed tests are unclear and available data not always used.	*Error* results are coded by the machine and should be documented. The .gxx files can provide this information and the types, reason, locations, and technicians associated with the error should be tracked.
	High failed test rates in certain projects.	*No result* test results are often caused by a power failure.
		*Invalid* results are not caused by testing a specimen of insufficient volume or one which contains saliva the way poor sputum quality is defined in a NTP. Rather, they seem to be caused by other problems with sputum. Emphasizing correct sputum collection techniques, including mouth rinsing to remove food or particles which could inhibit the assay, may help reduce *Invalid* results, as well as improve yield.
**Drug resistance**	Some confusion about clinical decisions after receiving rifampicin resistant and indeterminate results.	Patients with indeterminate results have TB, but rifampicin resistance cannot be confirmed due to a very low burden of TB bacilli in the specimen. Unless there is documented DR-TB risk, first-line treatment can be started. Follow-up of these patients is warranted.
	Trepidation over Xpert MTB/RIF use overburdening DR-TB programs.	The majority of drug-resistant cases in almost all countries will be found among new cases, requiring testing of people with suspected TB rather than patients already in TB treatment.
		Patients with rifampicin resistance will be detected in greater numbers and with greater speed than under current conditions, and significant resources and coordination will be required.
**Recording and reporting**	Supplied GeneXpert Dx software is not appropriate for analyzing patient cohorts, many times failed tests are recorded on paper with a generic error ‘result’, underreporting of errors, lack of clear guidance from national programs on recording and reporting of cases identified by Xpert MTB/RIF.	Dissemination and uptake of WHO guidelines on recording and reporting should be adopted by countries and their NTPs.
		Automated reporting mechanisms can improve both the timeliness and accuracy of reporting as well as assist in supply chain management.

The projects consumed on average 979 tests per four-module machine per year (range 675 in Moldova-1849 in Cambodia). Theoretically a 4-module machine could run 4,000 tests a year (four tests per module/day, and 250 working days a year). However, several factors impacting utilization were identified: the number of hours laboratory technicians were available to work (frequently only 4–5 hours per day), available power supply during the day, the number of people with suspected TB attending the facility, and the selectivity of the testing algorithm and case finding approach. For example, Cambodia only tested 4 days a week due to mobile clinic logistics and travel.

#### Algorithms and case finding approaches

A wide variety of diagnostic algorithms incorporating MTB/RIF testing were implemented (Table 3). Several projects employed multiple algorithms, though the majority used the MTB/RIF assay as a second test for patients with negative smear microscopy results. Some projects used active case finding strategies, others used a passive approach. Funding requirements of TB REACH focused all MTB/RIF testing on patients with suspected TB, as opposed to already-diagnosed patients suspected of having DR-TB. In practice however, a number of projects reported use of a small number of MTB/RIF tests as drug sensitivity test (DST) in specific cases. All projects tested pulmonary samples only, and while some projects accepted samples from children, very few children were tested due to difficulties in getting quality sputum samples.

**Table 3:** Overview of early Xpert MTB/RIF testing approaches in nine countries

**Country**	**Xpert MTB/RIF testing strategy**	**Point-of-care (POC) or referral for treatment**	**Laboratory process for requesting Xpert test**	**Time from initial screen to Xpert + result**	**Time from submission of sputum for Xpert MTB/RIF test to result**
Bangladesh	A - Direct to Xpert for all with history of previous TB treatment	POC	Automatic in laboratory	0-1 day	Same day
	B - Xpert following SS- results and TB Suggestive CXR				
Cambodia	Xpert following positive verbal and/or CXR screens	POC	Automatic in laboratory	0-1 day	0-1 day
DR Congo	Xpert following SS- results	Both, using a hub and spoke transport system to augment testing numbers	After follow up tests and review	7-10 days	1 day
Kenya	Xpert following SS- results	Both, using a hub and spoke transport system to augment testing numbers	Automatic in laboratory for those with negative smear results	1-4 days	1 day
Malawi	A - Direct to Xpert for all hospitalized patients with suspected TB	Both, using a hub and spoke transport system to augment testing numbers	Automatic in laboratory for those with negative smear results	1 day for walk in patients and up to 10 days for referral patients	0-3 days
	B Xpert following SS- results				
Moldova	Direct to Xpert; parallel smear microscopy	POC at most facilities; referral at AIDS Centers	Automatic in laboratory	0-1 days	0-1 days
Mozambique	Xpert following SS- results	Referral	Automatic in laboratory for those with negative smear results	Up to 7 days for referral patients	2 days
Nepal	Xpert following SS- results and a suggestive CXR	Both, using a hub and spoke transport system to augment testing numbers	After follow up tests and review	1 day for walk in patients and up to 10 days for referral patients	1-2 days
Pakistan	A - Direct to Xpert for all with history of previous TB treatment	POC	Automatic in laboratory	0-1 day	Same day
	B - Xpert following SS- results and TB Suggestive CXR				

The majority of projects used some sort of referral system to increase the numbers of people tested (Table 1). None of the projects systematically provided the MTB/RIF assay as a primary diagnostic test for people with HIV. Although many sites had ART clinics in their facilities, the majority of people tested were not from HIV centres, and the HIV status of those being tested with the MTB/RIF assay was often unknown.

#### Time to diagnosis

Table 3 presents an overview of the time needed for a patient to receive results of an Xpert MTB/RIF test. In four of the projects the intent was to provide results the same day and initiate treatment. This was generally implemented except when the number of samples exceeded machine throughput. Other projects used algorithms and/or referral systems that improved the standard turnaround time for bacteriological tests, but were not the same day. Turnaround times ranged from same day to over a week when a referral system was in place and/or the sample was transported from a remote facility. At sites that used referral systems, most testing was conducted on people presenting at the facilities rather than on sputum transported through referral systems. This diagnostic timeline still appeared to represent a shorter time to treatment for those with smear negative but MTB-positive results compared to initiative treatment as a smear negative case, but it was difficult to measure. In Moldova however, where parallel smear microscopy was conducted for all people with suspected TB, among those with MTB-positive results, only 56% were positive by smear microscopy. This led to immediate treatment for a large proportion of people who otherwise would have had to wait for CXR and/or culture results before treatment could begin.

In Cambodia and for hospitalized patients in Malawi, MTB/RIF tests were performed on the same day that sputum was submitted but, due to the number of individuals indicated for testing and laboratory work load, some had to wait to receive their results until the following day. In Pakistan, Bangladesh, Mozambique and Kenya, smear microscopy and, if indicated, MTB/RIF testing, were performed on a single sputum specimen submitted by each presumptive TB patient, shortening the time from sputum collection to MTB/RIF results. The DRC project reported a wide variance of times to diagnosis depending on the length of time it took to transport samples from peripheral microscopy laboratories. Malawi used a referral network including community sputum collection points and microscopy centres with two deliveries per week to augment the number of people tested. Mozambique also used a hub system, although systems to transport and process referred samples were difficult to maintain over time. Measures taken to reduce turn-around-time included using the same specimen to perform smear microscopy and the MTB/RIF test, training lab technicians to automatically conduct the MTB/RIF tests following smear-negative results (as opposed to waiting for a referral form), and paying for extra human resource support for laboratory staff.

### Preparation and installation requirements

#### Procurement and service

For all projects, procurement was done through the Global Drug Facility, with cartridges shipped in two or three batches to compensate for the short shelf life of the product in 2012 (8–12 months). The median lead time (order placement until delivery to port of entry) for ordering machines and test cartridges was 40 days (range 12–62). Shipping costs added to the test cost and ranged from 0.33 USD to 1.44 USD per test.

Following the cartridge price reduction in July 2012 from USD 16.68 to USD 9.98 [24], a number of shipments were delayed due to a surge in orders from countries that had postponed ordering to benefit from the price reduction. This resulted in a backlog of orders that could not be met by Cepheid’s production capacity at that time. In addition, a manufacturing problem lowered production capacity in the Winter and Spring of 2012–13 [25]. As of July 2013, the cartridge shortage issue had been resolved.

Cepheid has agreements with local service providers in different countries which are intended to assist in procurement, service and repair needs. The experience of projects with these providers was quite varied and some local providers were found to be severely inadequate. As increasing Xpert MTB/RIF testing occurs, the demand for competent in-country services will also increase. Identifying competent and responsive local providers will be necessary for scale up efforts.

#### Training/human resource issues

Many projects mentioned that when placing machines in more peripheral sites, training in how to use a computer was more difficult than training for the test procedure itself. Several also reported that the lack of multi-language settings for the GeneXpert Dx software was a challenge, particularly in settings where the Roman alphabet is not used. Implementers employed a range of people to manage GeneXpert equipment, including lay workers, TB staff, general laboratory staff and new project hires. Prior laboratory experience is not required to competently use the assay (Table 2). Pakistan, which employed lay individuals to perform testing, had one of the lowest failed test rates of the projects (Table 4). All projects reported high levels of laboratory staff satisfaction with the machine, its ease of use, the ability to complete other tasks while testing, and rapid test results.

**Table 4:** Result data from first 12 months of Xpert MTB/RIF implementation in nine countries

**Xpert site**	**Tests**	**MTB Positive**		**Rifampicin**						**Failed Tests**		**Type of failed test**						**MTB + w/o failed tests**
		**N**	**%**	**Sensitive**		**Indeterminate**		**Resistant**		**n**	**%**	**Error**		**Invalid**		**No result**		
				**n**	**%**	**N**	**%**	**n**	**%**			**N**	**%**	**n**	**%**	**n**	**%**	
Bangladesh	1428	286	20.0%	249	87.1%	11	3.8%	26	9.1%	99	6.9%	85	85.9%	10	10.1%	4	4.0%	21.5%
Cambodia	3697	768	20.8%	741	96.5%	22	2.9%	5	0.7%	439	11.9%	169	38.5%	264	60.1%	6	1.4%	23.6%
DR Congo	6348	567	8.9%	498	87.8%	17	3.0%	30	5.3%	1032	16.3%	868	84.1%	107	10.4%	57	5.5%	10.7%
Kenya	2803	258	9.2%	238	92.2%	11	4.3%	9	3.5%	266	9.5%	240	90.2%	23	8.6%	3	1.1%	10.2%
Malawi	6258	632	10.1%	602	93.5%	9	1.4%	21	3.3%	853	13.6%	546	64.0%	265	31.1%	42	4.9%	11.7%
Moldova	11472	1965	17.1%	1227	62.4%	48	2.4%	690	35.1%	673	5.9%	387	61.7%	105	15.6%	181	26.9%	18.2%
Mozambique	6823	910	13.3%	826	90.8%	18	2.0%	66	7.3%	871	12.8%	521	59.8%	216	24.8%	134	15.4%	15.3%
Nepal	6943	1376	19.8%	1257	91.4%	14	1.0%	105	7.6%	744	10.7%	409	55.0%	148	19.9%	187	25.1%	22.2%
Pakistan	2201	433	19.7%	390	90.1%	13	3.0%	30	6.9%	130	5.9%	77	59.2%	37	28.5%	16	12.3%	20.9%
**All Countries**	**47973**	**7195**	**15.0%**	**6028**	**83.8%**	**163**	**2.3%**	**982**	**13.6%**	**5107**	**10.6%**	**3302**	**64.7%**	**1175**	**23.0%**	**630**	**12.3%**	**16.8%**
**South Africa**^**a**^	**1180669**	**171792**	**14.6%**	**155811**	**90.7%**	**2443**	**1.4%**	**12266**	**7.1%**	**32561**	**2.8%**							**15.0%**

^a^Reference 26. Data for South Africa is presented as a comparison after 25 months of implementation.

The programmatic aspects of implementation, including clinical decisions on testing, completion of MTB/RIF request forms, transportation of specimens, and registering results and treatment decisions, especially in cases of rifampicin resistant results, required the most training.

#### Infrastructure and power supply

In most cases, local infrastructure improvements to address issues of climate control, work space security, ventilation, and dust control were required to set up GeneXpert equipment. Procurement needs included air conditioners or heaters, fans, safety equipment, and in a couple of instances, refrigerators for stored samples. The main improvement required was for uninterrupted power supply to complete each test. Although the manufacturer offers an uninterrupted power supply (UPS) device for each GeneXpert machine, additional protection is often needed as said UPS was developed for high-income settings with relatively stable power supplies. In Cambodia, a generator was used for machines in mobile clinics (Additional files 8,9). Many projects used locally sourced power solutions including inverters and large backup batteries at costs comparable to or lower than the UPS supplied by the manufacturer (300–900 USD). See Table 2 for recommendations on UPS specifications and Additional files 5,6,7,8 for images. The laptop’s built-in battery drains the UPS less quickly than a desktop and may result in fewer failed tests; however, laptops may be more vulnerable to theft. The four-module desktop machine costs 17,000 USD (500 USD less than a laptop unit).

## Test results

### Overall results

Over the first 12 months of implementation the nine projects consumed 47,973MTB/RIF tests (range 1,428-11,472). MTB/RIF testing resulted in a positive test for 15.0% (7,195) of all cartridges used and for 16.8% of all valid tests (excluding failed tests). Positivity among valid results varied between 10.1% and 23.6% (Table 3). Due to the wide variety of diagnostic algorithms used by the different projects, it is difficult to directly compare yields across sites. The yield of TB cases at sites utilizing CXR as a screening test and/or using Xpert MTB/RIF as the first test (Bangladesh, Cambodia, Moldova, Pakistan) was 50-70% higher than sites which went straight from smear microscopy to the MTB/RIF test (Kenya, Mozambique, DRC). The overall positivity in these 9 projects was similar to yields from the South Africa’s rollout of Xpert MTB/RIF (15%) [26]. Among all MTB-positive results, 2.3% (163) were RIF indeterminate. Unless there was some DR-TB risk, first-line treatment was started on patients with RIF-indeterminate results and a repeat MTB/RIF test was not performed.

### Failed tests

The overall failed test rate was 10.6% (range 5.9% in Moldova and Pakistan to 16.3% in the DRC). When data was pulled directly from the GeneXpert machine as opposed to the laboratory registers, a number of unreported failed tests were discovered. It appeared that technicians may have re-tested specimens until a positive or negative result was achieved, and reported only the final result. More than one quarter of failed tests were directly related to power issues, either through *No Result* outcomes or 2127 *Errors* but varied greatly by country.

A common test-related error was that of signal loss associated with an earlier version of MTB/RIF cartridges (G3 vs G4). Signal loss errors made up 44% of all errors in Nepal and 28% in DRC, but only 11% in Pakistan (data not shown) and appeared to be related to specific batches of G3 cartridges rather than a general problem. In Kenya, *Error* rates were much higher among G3 tests (12.9% vs 5.2%), but in other countries there were a higher proportion of *Error* results among the G4 cartridges. *Error* results made up the majority of the failed tests (64.7%), however in some sites *No Result* and *Invalid* findings contributed significantly to the failed test results. See Table 2 for descriptions and recommendations concerning failed tests. We were unable to accurately track the exact rates of retesting among the projects as laboratories attempted to use the same sputum sample, but with referred samples follow up was often difficult and this information was not well documented.

The overall module failure rate in the projects was 42% (77/182), but in Moldova only 7% (5 of 68) modules needed replacement while in Mozambique, Nepal and Malawi more than half needed to be replaced. In Bangladesh and Pakistan all modules needed to be replaced. This was not just calibrated modules but overall failure. Many module problems seemed to be clustered suggesting that other implementation issues, such as irregular power supply and currents, dust build-up, overheating, and staff quality control may have contributed to the failures.

### Rifampicin resistance

Among all valid tests, 2.3% of results showed rifampicin resistance, (0.9% excluding Moldova). Overall, 13.6% (n = 982) of MTB-positive results were resistant to rifampicin, with Moldova accounting for 69.5% of those cases and Nepal accounting for 10.6%. Excluding Moldova, 5.6% of MTB-positive results were resistant to rifampicin. Projects referred the patients to the MDR-TB treatment sites but did not generally provide active follow-up as TB REACH does not provide any funding for drug resistance treatment or support. Complete data on follow-up culture and DST were not available for many of the projects as almost all samples were sent to distant referral laboratories. Projects in Mozambique, DRC, Malawi and Nepal noted important barriers in getting DST results due to distance, and overworked laboratories. Most noted significant loss to follow-up of patients having to travel long distances though it was not quantified. Algorithms for treatment of people with a rifampicin resistant result varied. In settings where there are high levels of DR-TB, such as Moldova, any patient with a rifampicin resistant result was placed on a standardized second line treatment regimen immediately, reducing the treatment delay significantly over the previous system of consolidating samples and follow-up DST. In settings where drug resistance was less prevalent, confirmatory DST was requested before patients started second line regimens. Most patients who had a prior treatment history were able to initiate second line treatment immediately. Patients with identified rifampicin resistance through MTB/RIF testing would most likely not have been diagnosed with DR-TB in the absence of such testing until they had failed treatment or relapsed, as the projects targeted people with no history of TB and with limited access to conventional DST methods.

### Recording and reporting

All of these nine implementers were among the first to use MTB/RIF testing in their country and/or were the first to use it in a programmatic setting. When NTP reporting guidelines for MTB/RIF testing existed, they were often for diagnostic algorithms focused on detecting drug resistance, and were inadequate for these case-finding interventions. The projects in Cambodia and DRC were run by national and provincial TB programs respectively and had the ability to set recording guidelines. Cambodia records all MTB-positive results as smear-positive, while in DRC and Mozambique they are recorded as smear-negative. In other countries, reporting procedures had to be discussed with the NTP and the lack of global guidance on the issue at the time of testing created some confusion. In Pakistan, MTB-positive patients were at first reported as smear-positive, but following changes in NTP guidelines, they were subsequently reported as smear-negative. In Nepal, patients detected on the MTB/RIF assay were reported as smear-negative, but when patients had MTB-positive results with no CXR result, treatment was initially delayed due to confusion around guidelines. Malawi had no national guidelines for reporting MTB/RIF results, leading to variable practices among districts until implementing partners developed standard guidelines.

Projects took advantage of the fact that all machines are coupled with a computer to develop electronic registers, but there was general agreement that the pre-installed GeneXpert Dx software is not useful for capturing patient cohort information due to test data being individually processed with limited collating and exporting options. All projects used different locally developed databases to track tests and patients. Most projects enforced strict prohibitions on the use of external drives with the computers due to virus and malware threats. Data were downloaded from the machines via blank CDs or abstracted through automated reporting systems. DRC, Kenya, Pakistan and Bangladesh employed systems that provided immediate reporting of test results to the providers and referral systems over a GSM network.

## Discussion and conclusions

The findings of this study begin to fill the gaps among guidelines, research findings, and real-world implementation of MTB/RIF testing, where NTPs and their partners work to test people with suspected TB and drug resistance. The projects documented here had a wide range of experiences, approaches and results, demonstrating the versatility of Xpert MTB/RIF, but also highlighting a number of barriers to implementation and possible solutions. As others have pointed out, the technology has great potential, but also has important limitations. This study begins to address some of the concerns delineated through implementation experiences in the field. These projects have showed that with proper support for procurement, infrastructure and supervision, Xpert MTB/RIF can be deployed at or very close to the point of care in many settings initially considered difficult because of the hurdles to overcome [26]. Our findings generally coincide with other early assessments of the challenges and benefits of Xpert MTB/RIF implementation where many barriers can be surmounted if proper systems are in place and a coordinated country approach is taken with the best interests of the patient in mind [27].

Xpert MTB/RIF testing detected a large number of people with TB that routine services failed to detect. Data from South Africa shows similar results for MTB-positive yield but much lower error rates [28] although many of the projects described here placed the machines in different types of facilities, including mobile units and primary care facilities in challenging settings. When available, the use of CXR as a highly sensitive screening tool should be considered before testing with Xpert MTB/RIF, to save cartridges and increase yield [2,29]. Placing smear microscopy ahead of the MTB/RIF test in the diagnostic algorithm often resulted in lower yields compared with specimens tested directly on the MTB/RIF assay, as smear-positive patients were then excluded. However, the underlying prevalence of TB in the screened population, as well as the quality of smear microscopy services [30], play important roles in influencing yields among smear-negative specimens. Xpert MTB/RIF testing on smear-negative individuals, the general approach used here, expends significantly more resources and time, as all individuals are tested using smear microscopy and 80-90% of them are re-tested with Xpert MTB/RIF. New WHO guidance recommends that Xpert MTB/RIF replace smear microscopy when resources allow [31]. All of the projects described here had external funding for Xpert MTB/RIF testing and so it is curious that only a few used it to replace smear, but this was many times limited by national policy.

Tracking the types, codes, reason, locations, and technicians associated with failed tests is important for users to improve performance. There are generally three possible types of failed tests for MTB/RIF: human, power supply and test-related. Human errors generally arose from inadequate sputum collection or specimen preparation and can be tracked by their numeric code [32]. Automated reporting mechanisms can improve both the timeliness and accuracy of reporting errors and all results as well assist in supply chain management [33]. Some projects had very high rates of *Invalid* results which seem to be caused by problems with the quality of the sputum sample and proper mouth cleansing techniques. Better coaching from staff may be able to help in these instances. While the failed test rates are much higher than in South Africa, they are similar to some other larger early implementer multi-country initiatives [34]. Most of the projects placed the machines at lower level facilities which presents more of a challenge for implementation and supervision. An early experience in South Africa using mobile testing had comparable failed test results, although most were power supply related. [Personal Communication Liesl Page-Shipp] *No result* outcomes are often linked to power supply problems. In environments with irregular and unpredictable power, a backup power supply with an inverter should be capable of supporting the GeneXpert machine and computer for at least 2 hours in order to finish any test begun before a power failure (see Table 2). When GeneXpert machines are placed in facilities with other equipment needing power supply, generators are likely to be available and backup power may only be needed until the generator can be started. Ensuring timely calibration of the GeneXpert machines will also reduce errors. If the calibration is done after the module warranty expires and the module must be replaced, a new module or an extended warranty must be purchased. Modules can still fail outside of calibration and extended warrantees are available and should be purchased to protect the users.

As Xpert MTB/RIF provides both TB and rifampicin resistance results, it is important to weigh the benefit of early detection of drug resistance against the concern that large increases in DR-TB detection would overwhelm advanced diagnostic facilities and nascent drug-resistant TB treatment programs. Focusing MTB/RIF testing solely on DR-TB suspects is unlikely to produce large yields as the numbers of failures and retreatment cases at a district level is quite small in most settings. Furthermore, the majority of DR-TB cases will be found among new cases [35], requiring testing of people with suspected TB rather than patients already receiving TB treatment if meaningful MDR-TB treatment scale up is to take place. The application of algorithms focused on people with suspected TB will generally result in large increases in drug sensitive cases detected and a lower proportion of rifampicin resistant results. Nevertheless, DR-TB patients will be detected in greater numbers and with greater speed than under current conditions, and significant resources and coordination will be required for treatment, especially if MTB/RIF testing is to be done in areas without DR-TB treatment capacity as was the case in many of these projects. These projects were limited given funding was not provided for DR-TB treatment and active follow-up of rifimapicin resistant patients was generally not conducted and data is limited. However, in many countries, overall donor funding for DR-TB treatment remains unused as many people with DR-TB have not been diagnosed; so opportunities for expansion exist. Given the concern about slow expansion of MDR-TB treatment globally, South Africa has clearly shown that huge gains in detection and notification are possible in a short period of time using Xpert MTB/RIF as the primary diagnostic tool [1].

Apart from testing approaches and results, there were a number of other implementation issues that merit discussion. Although many point of care testing strategies focus on finger prick or dipstick testing, a recent review discussed the positioning of diagnostic tests in the health system and argued that many tests could be considered point of care, including smear microscopy and MTB/RIF, if the test and treat cycle could be completed in the same clinical encounter (or the same day) [36]. However, testing algorithms and work-flow practices often gets in the way of providing quick results to the patient and ensuring same-day TB treatment and this was seen in a number of projects [36]. While Xpert MTB/RIF testing can provide highly sensitive results quickly in a number of different settings, the health system must provide a supportive environment; tightly controlling the machine and testing in high level laboratories may sometimes be achieved to the detriment of the patient. A recent study has made a strong argument for the use of MTB/RIF as a point-of-care test showing a reduction in delay, empiric treatment, and the ability of nurses to implement the test in primary care settings [37].

Decisions on machine location should be given careful consideration with respect to expected numbers of people tested and the need for referral systems in addition to power supply. Although many peripheral health facilities do not have the infrastructure to handle complicated testing [38], the main limitations we identified for decentralized MTB/RIF testing - algorithms, power, temperature and numbers of tests expected - can all be overcome with proper support, although in some remote areas supervision is difficult and seemed to complicate performance.

Training staff to use GeneXpert equipment was generally described as straightforward, supporting views of other early implementers [39]. However, the need for retraining and supervision cannot be overlooked as staff turnover is a constant reality. As part of training, ensuring solidified national testing and treatment guidelines are in place is critical. Country adoption of the WHO policy guidance around testing algorithms [2] as well as recent WHO new case and outcome definitions incorporating MTB/RIF and other WHO recommended tests[40], will greatly help standardize the process. All people with MTB/RIF positive results should be recorded as bacteriologically positive cases. The adoption of WHO recommended strategies and reporting systems will reduce the inconsistencies that the early implementers faced, though it must be acknowledged that field adoption of new guidance often takes time. Delays in customs clearing, in country transportation and other preparatory activities, such as training, will shorten the effective usage time for the cartridges. Staggering orders can help avoid expiry of cartridges, which have a short shelf life.

The technology has the possibility of being quite disruptive to national testing and treatment policy, and to recording and reporting forms and training. With donors including PEPFAR and The Global Fund beginning to fund the expansion of MTB/RIF testing in many countries, lessons learned from pilot projects that can be applied to a large expansion of testing must be disseminated. One of the main areas of concern about MTB/RIF testing is the additional cost to country diagnostic budgets which these projects did not have to address as the work was grant funded, therefore this experience cannot directly address or inform those concerns. Just as a few countries fund their own second line treatment programs, we feel that the benefits of improved diagnostics for TB and drug-resistant TB provide ample justification for long term support of improved diagnostics from donors. This study is not representative of all early implementer experiences with MTB/RIF testing but rather provides an overview of the shared issues across countries and of many different approaches to MTB/RIF implementation on a programmatic basis. Further programmatic studies are needed on issues such as case notification, time to diagnosis, cost effectiveness of MTB/RIF use in case finding programs and use at the most peripheral of health units (point of care testing) - all issues that our work was not designed to answer.

The experiences of these early implementers show that a variety of MTB/RIF testing approaches can be successfully employed across many different settings. This early information can be usefully applied in the on-going global scale up of MTB/RIF. The MTB/RIF test has limitations, but also provides an excellent opportunity to improve the way TB and drug-resistant TB are diagnosed on a wide scale.

## Competing interests

Jacob Creswell and Suvanand Sahu are members of the TB REACH secretariat and Lucica Ditiu is the Executive Secretary of the Stop TB Partnership, but were not involved in the initial projects design or the decision to fund any of the projects.

## Authors’ contributions

JC, AJC, EA, SS and LD conceived of the study design. AJC, EA, MM, AB, EJC, RPY, AM, and BR, supervised the testing and data collection in countries. LB and MB were responsible for monitoring and evaluating data collection. JC, AJC and MB drafted the manuscript. All authors interpreted the data and reviewed the manuscript. All authors read and approved the final manuscript.

## Pre-publication history

The pre-publication history for this paper can be accessed here:

http://www.biomedcentral.com/1471-2334/14/2/prepub

## Supplementary Material

Additional file 1 Xpert equipment in USAID AMPATH Mycobacteriology Reference Laboratory, Moi Teaching and Referral Hospital, Eldoret, Kenya. Click here for file

Additional file 2 Busia District Hospital, USAID AMPATH Reference lab, Busia, Kenya. Click here for file

Additional file 3 Working in a private laboratory in Dhaka, Bangladesh. Click here for file

Additional file 4 Training for Xpert MTB/RIF in Nepal. Click here for file

Additional file 5 Two linked 4-module Xpert machines with backup power supply in Zomba Hospital, Malawi. Click here for file

Additional file 6 CIDASA primary health center in Bukavu, South Kivu Province, DR Congo. Click here for file

Additional file 7 Shabunda Reference Hospital, South Kivu Province, DR Congo. Click here for file

Additional file 8 Cambodia mobile clinics showing GeneXpert machine connected to a generator. Click here for file

Additional file 9 Cambodia mobile clinics showing CXR screening powered by a generator in truck outside window. Click here for file
