# Supplementary figures and images for: Results from early programmatic implementation of Xpert MTB/RIF testing in nine countries
Source: BMC Infect Dis. 2014 Jan 2;14:2. doi: 10.1186/1471-2334-14-2 (PMC3898850; doi:10.1186/1471-2334-14-2)

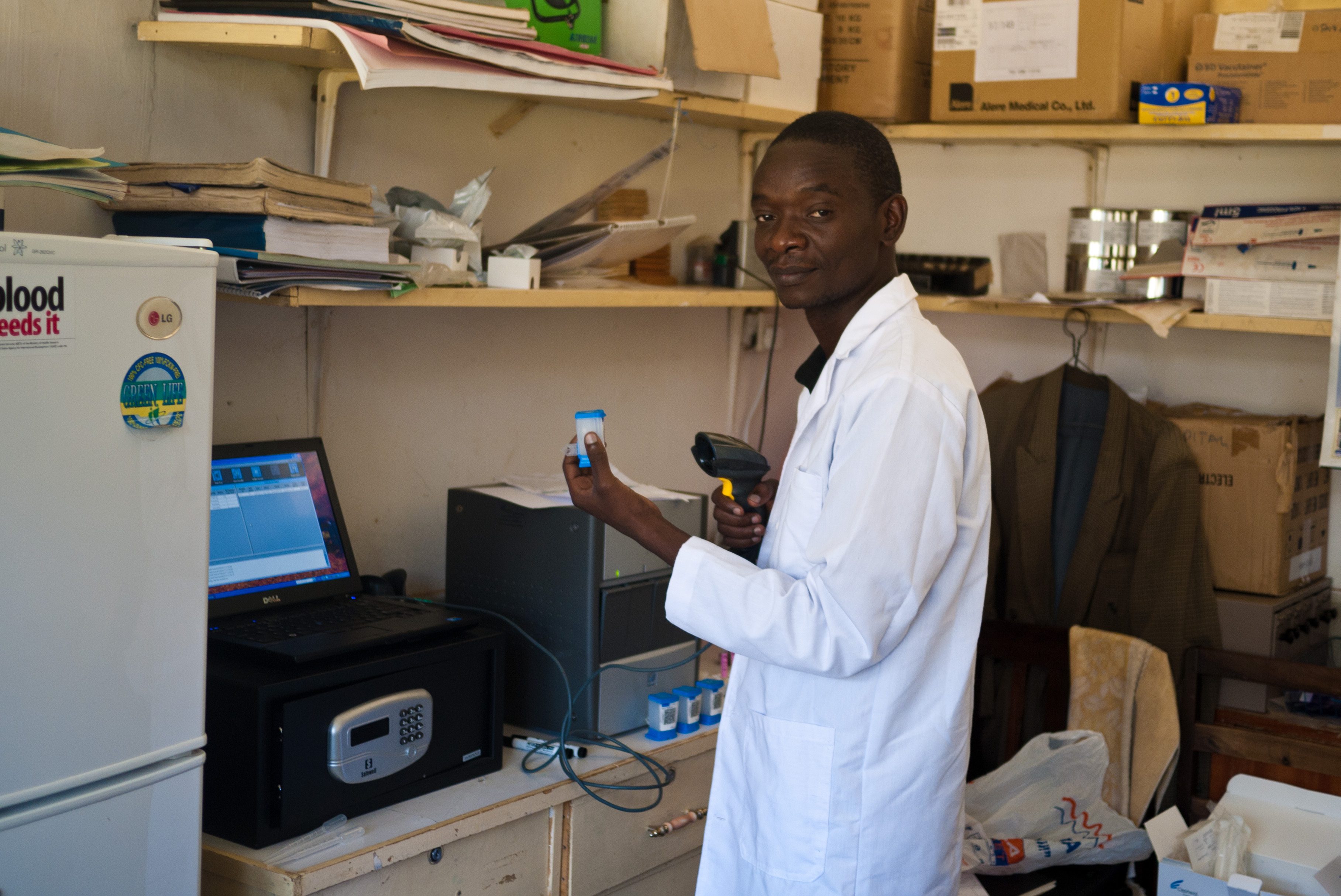

Supplement: Additional file 1 — Xpert equipment in USAID AMPATH Mycobacteriology Reference Laboratory, Moi Teaching and Referral Hospital, Eldoret, Kenya. [file 1471-2334-14-2-S1.jpeg]

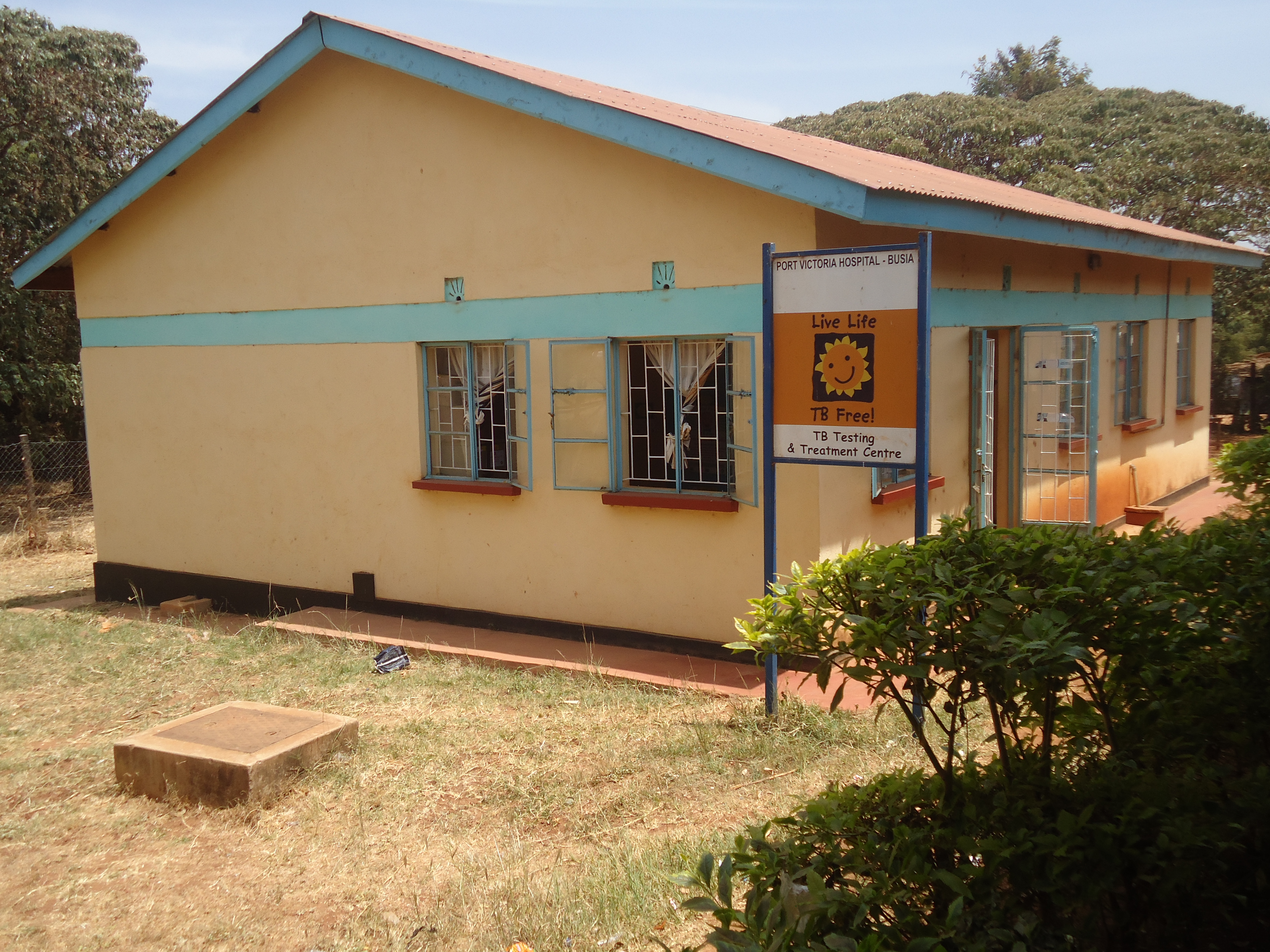

Supplement: Additional file 2 — Busia District Hospital, USAID AMPATH Reference lab, Busia, Kenya. [file 1471-2334-14-2-S2.jpeg]

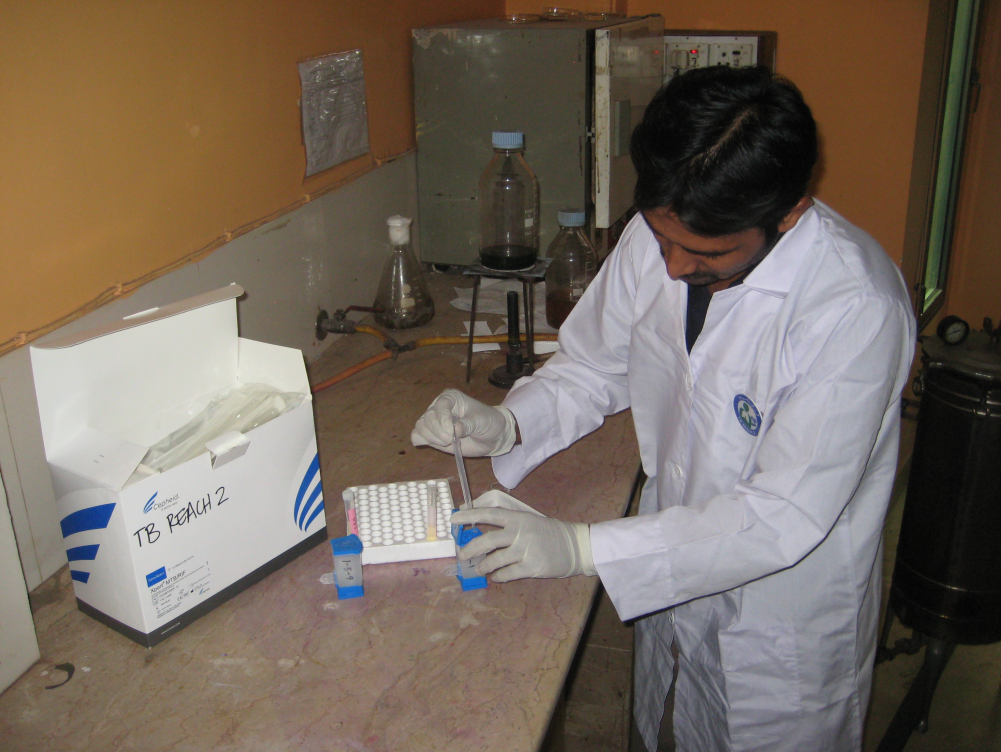

Supplement: Additional file 3 — Working in a private laboratory in Dhaka, Bangladesh. [file 1471-2334-14-2-S3.png]

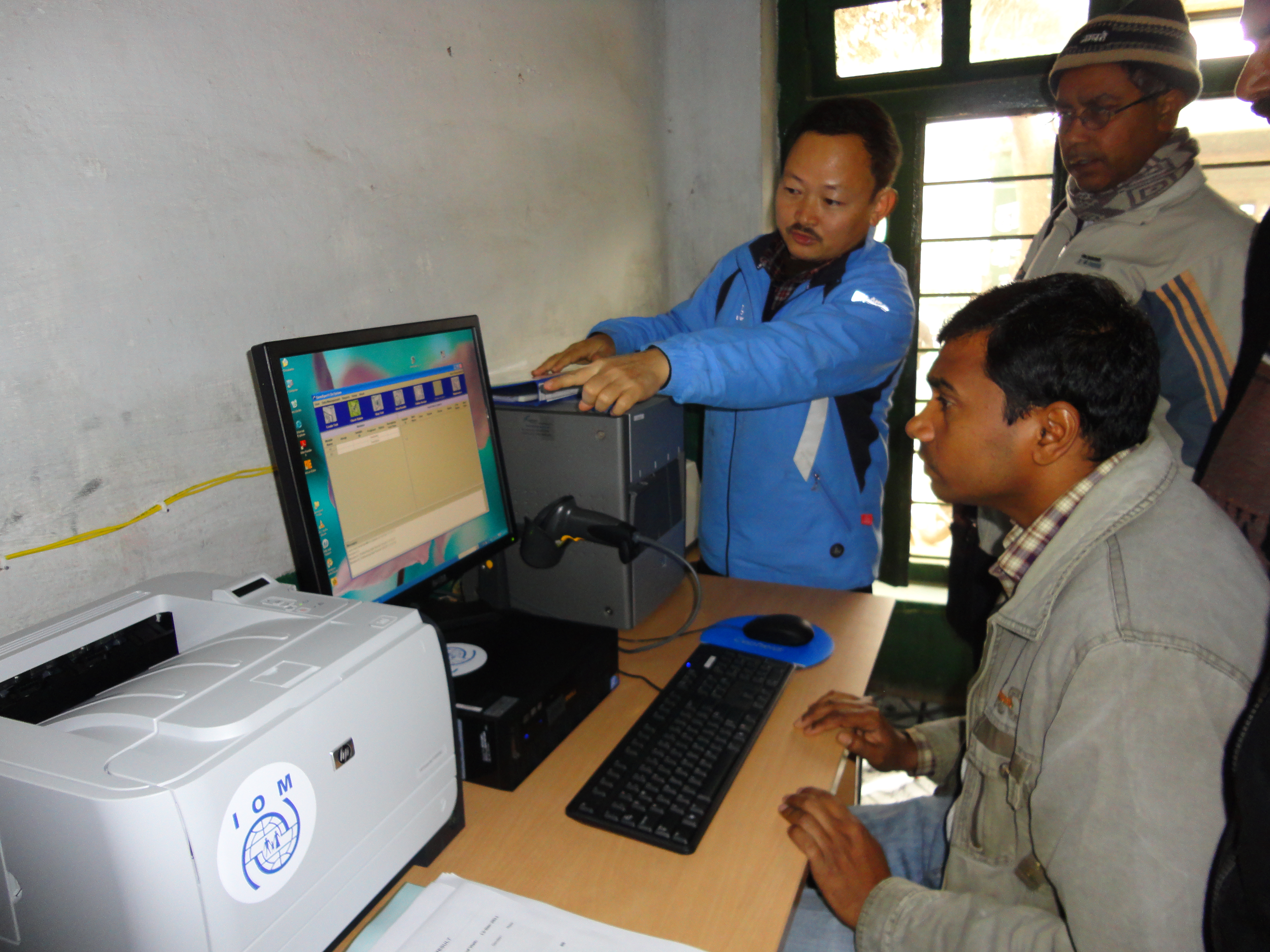

Supplement: Additional file 4 — Training for Xpert MTB/RIF in Nepal. [file 1471-2334-14-2-S4.jpeg]

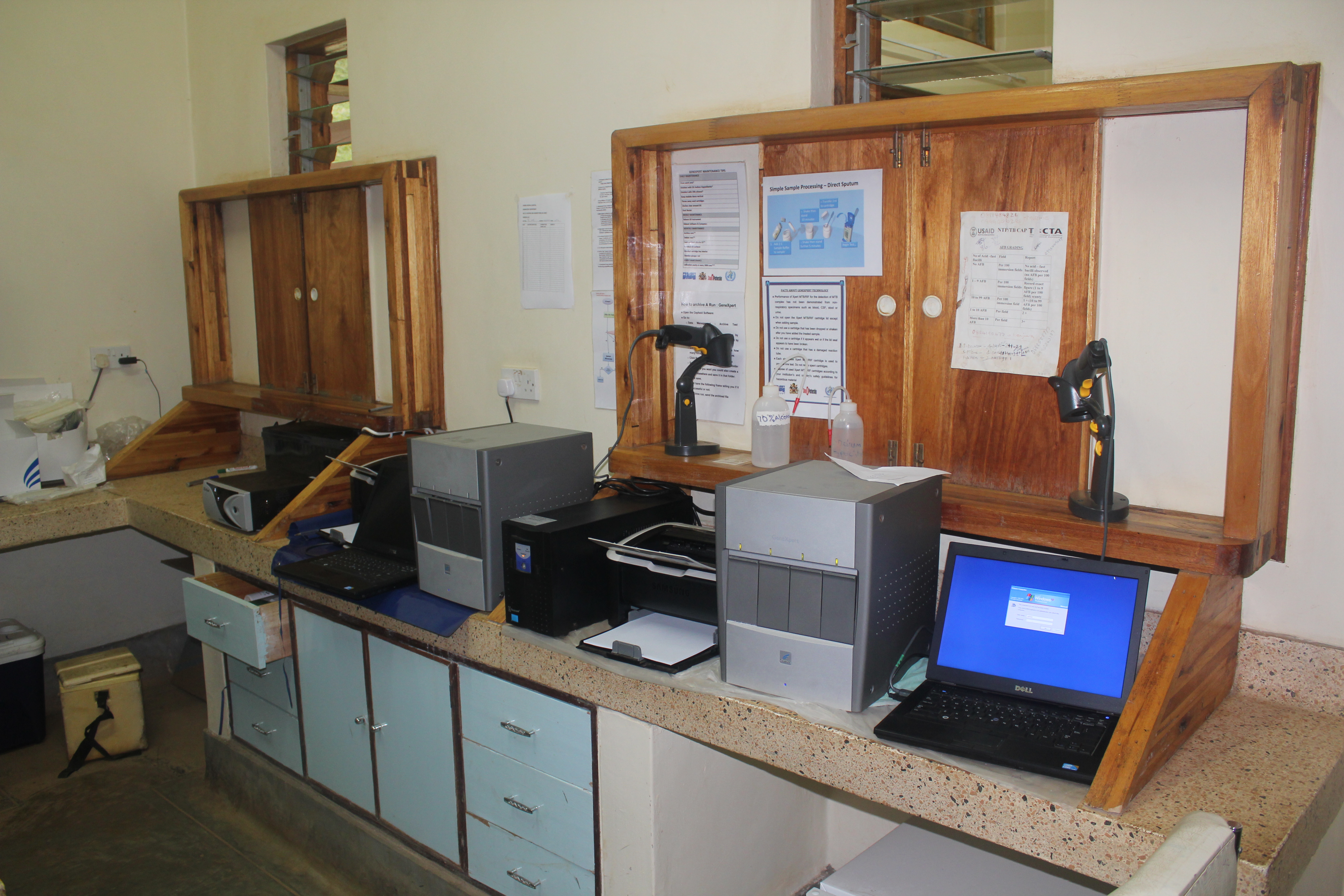

Supplement: Additional file 5 — Two linked 4-module Xpert machines with backup power supply in Zomba Hospital, Malawi. [file 1471-2334-14-2-S5.jpeg]

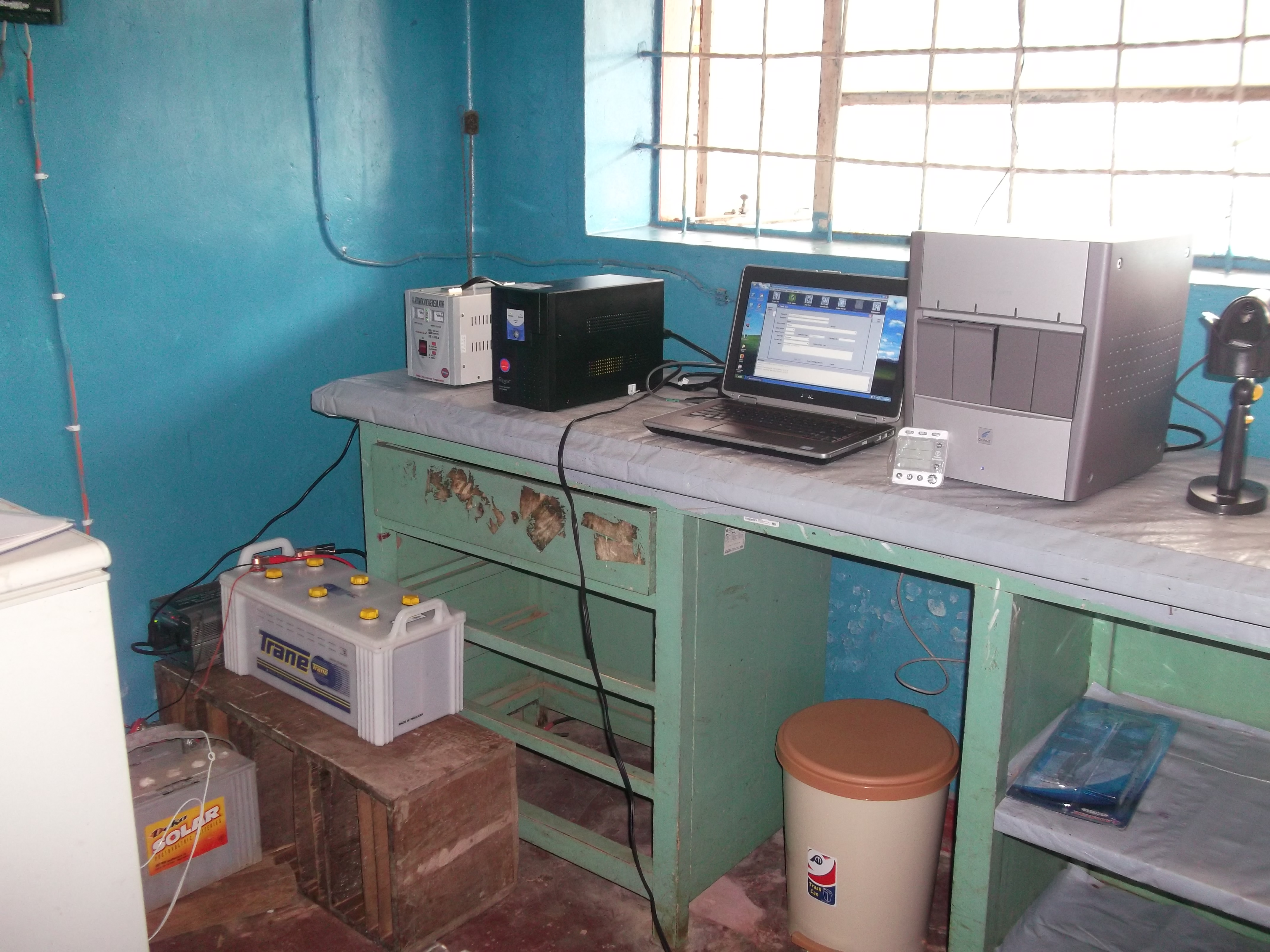

Supplement: Additional file 6 — CIDASA primary health center in Bukavu, South Kivu Province, DR Congo. [file 1471-2334-14-2-S6.jpeg]

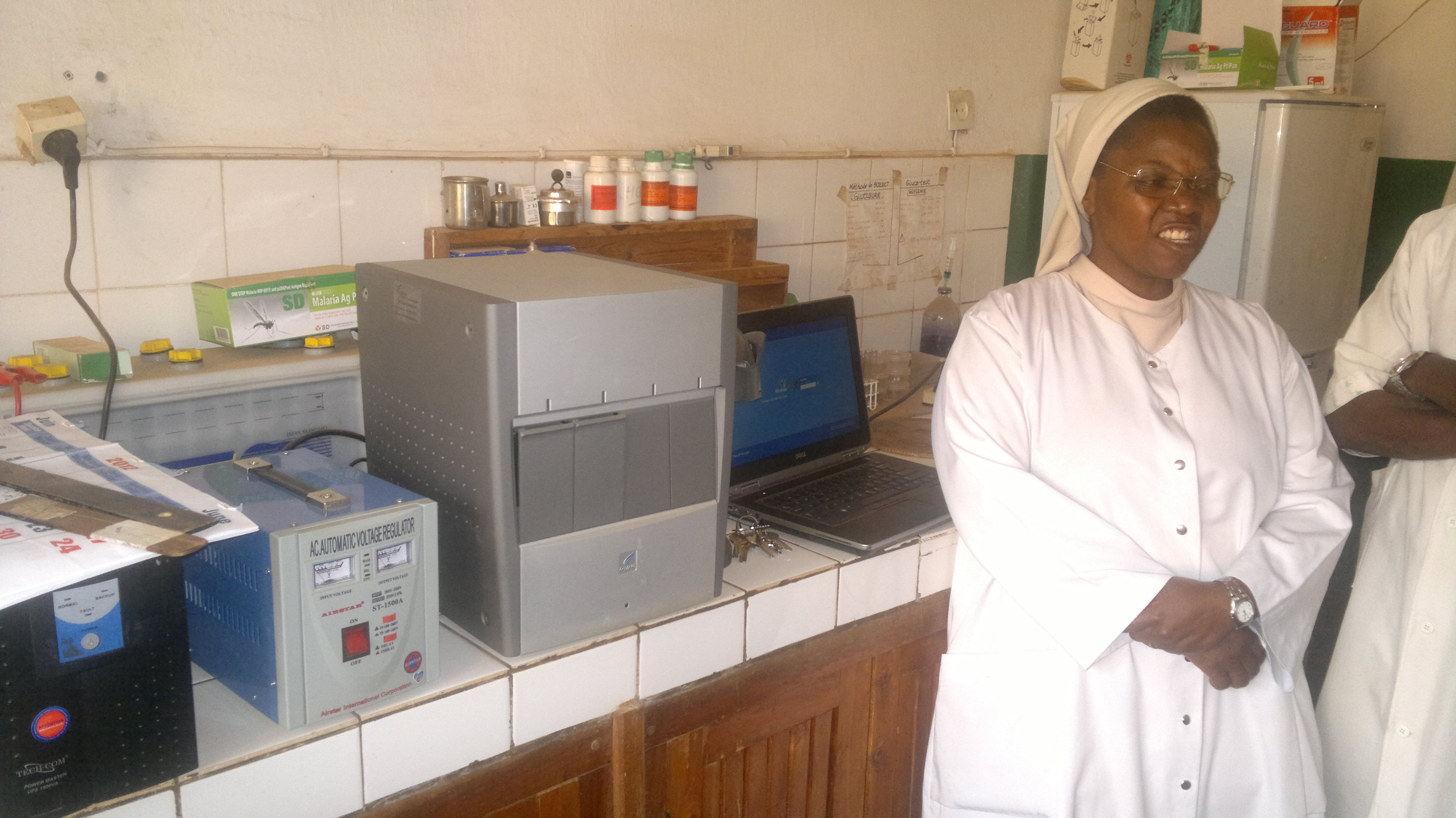

Supplement: Additional file 7 — Shabunda Reference Hospital, South Kivu Province, DR Congo. [file 1471-2334-14-2-S7.jpeg]

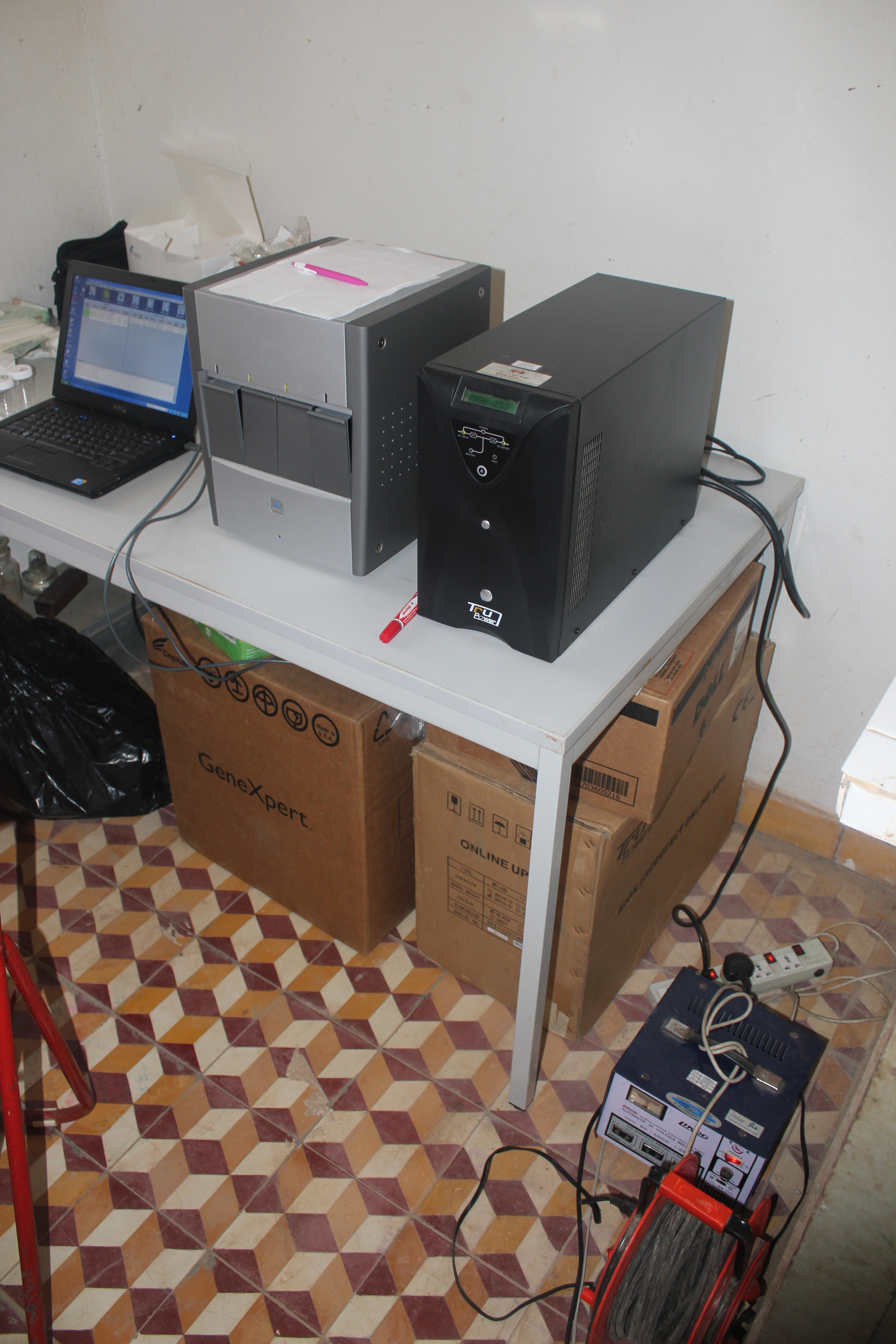

Supplement: Additional file 8 — Cambodia mobile clinics showing GeneXpert machine connected to a generator. [file 1471-2334-14-2-S8.jpeg]

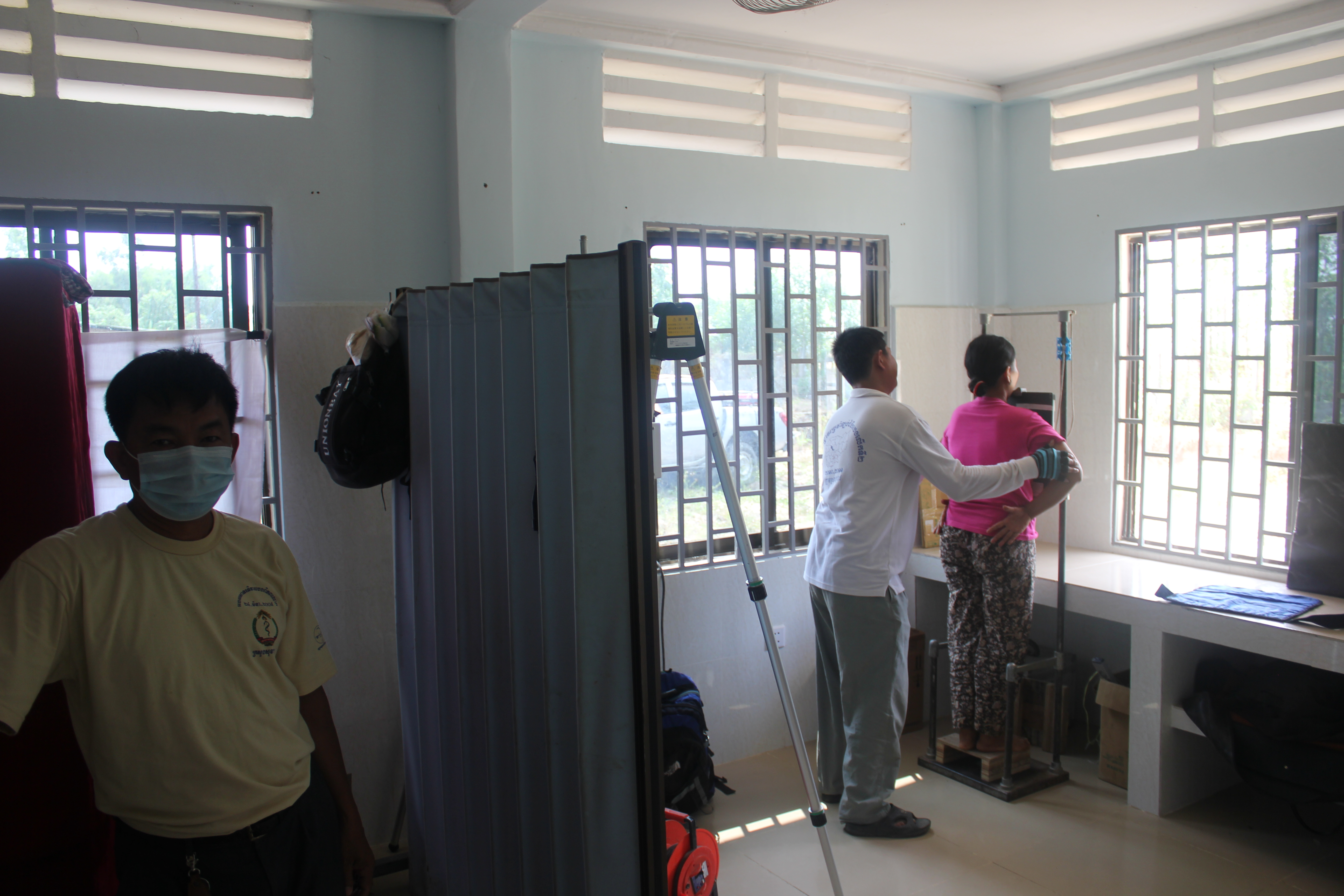

Supplement: Additional file 9 — Cambodia mobile clinics showing CXR screening powered by a generator in truck outside window. [file 1471-2334-14-2-S9.jpeg]
